# Neuro-Oncology During the COVID-19 Outbreak: A Hopeful Perspective at the End of the Italian Crisis

**DOI:** 10.3389/fmed.2020.594610

**Published:** 2020-11-27

**Authors:** Matteo Simonelli, Enrico Franceschi, Giuseppe Lombardi

**Affiliations:** ^1^Department of Biomedical Sciences, Humanitas University, Milan, Italy; ^2^Humanitas Cancer Center, Humanitas Clinical and Research Center — IRCCS, Milan, Italy; ^3^Department of Medical Oncology, Azienda USL/IRCCS Institute of Neurological Sciences, Bologna, Italy; ^4^Department of Oncology, Oncology 1, Veneto Institute of Oncology IOV-IRCCS, Padua, Italy

**Keywords:** neuro oncology, COVID-19, gliomas, brain tumors, SARS–CoV−2

Since December 2019, when severe acute respiratory syndrome coronavirus 2 (SARS-CoV-2) had been identified for the first time in Wuhan, Hubei, China, the outbreak has quickly become a worldwide pandemic with disruptive health, social, and economic impact. As of August 2020, more than 21,600,000 cases and 775,000 confirmed deaths have been reported by the WHO across all continents, with exponential spread initially in Europe, and currently in the East, United States, and Latin America. Italy has been hurt dramatically, being the first European country involved, and the epicenter of the pandemic for a few months (1). The regions of northern Italy (Lombardy, Veneto, Emilia-Romagna, and Piedmont) were particularly affected, requiring immediate and tight emergency measures to contain the infection followed by a national lockdown set since the beginning of March (2). In this complex scenario, the Italian cancer community have faced many arising tough challenges (3). Cancer patients are more susceptible to infections because of their immunosuppressive status and, at least in theory, at major risk of developing severe complications from the coronavirus disease (COVID-19), including adult respiratory distress syndrome (ARDS), intensive care unit admission, and even death. The first reports from China seem to confirm this hypothesis suggesting that patients with cancer are more likely to contract the virus and to develop COVID-19-related complications (4). More recent works indicated with strong evidence that cancer patients are at an increased risk of mortality and severe illness due to SARS-CoV-2 infection, regardless of whether they have active cancer, are on anticancer treatment, or both (5, 6). A recent multicenter European study involving 890 cases with confirmed COVID-19 demonstrated a worsening gradient of mortality from breast cancer to hematological malignancies and identified male gender, older age, and number of comorbidities as negative prognostic variables (7). Patients diagnosed with primary brain tumors (PBTs) are considered one of the most fragile and vulnerable category due to several factors: the older age along with the multiple age-related frailties and comorbidities, the frequent presence of neurological deficits causing loss of autonomy in the activities of daily living and increased risk of thromboembolic events, the often severe lymphopenia both disease and treatment-related (e.g., alkylating agents such as temozolomide and nitrosourea), and finally the chronic use of steroids to control brain edema leading to further immunosuppression and to an increased susceptibility to infection (8). Moreover, preliminary clinical data suggest that the SARS-CoV-2 infection can commonly involve the central nervous system (CNS) especially in those patients with lower lymphocyte counts, causing neurological manifestations both central such as dizziness, headache, acute cerebrovascular disease, depressed level of consciousness, ataxia, and seizures, or peripheral such as hypogeusia, and hyposmia (9). The first reports from autopsies of patients with COVID-19 revealed that the brain tissue was often hyperemic and edematous, with some areas of neuronal degeneration (9). As for other coronaviruses, including SARS-CoV and MERS-CoV, the main pathogenic mechanism may be the direct CNS invasion of SARS-CoV-2. The olfactory nerve has been recently described as the potential neural pathway used by the virus to gain entry into the CNS (10, 11). In addition, the SARS-CoV-2 virus seems to exploit the angiotensin-converting enzyme 2 receptor to entry inside the cells, and these receptors have been detected into the brain over glial cells and neurons (12). Another pathogenic mechanism under current investigation potentially leading to neurological damages in patients with COVID-19 is represented by endothelial ruptures in cerebral capillaries followed by bleeding within the brain parenchyma (12). For all these reasons, it is almost conceivable that PBTs patients, with an already injured brain, could be more exposed in terms of frequency and seriousness to these symptoms. In the context of the COVID-19 pandemic, the need for close and continuous assistance by caregivers, usually family members, could increase exponentially the risk of interfamily infection. Moreover, travel restrictions imposed to mitigate the SARS-CoV-2 spread limit the possibility of patients to move around the country, the attendance for repeat appointments, and continuity in care in the referral centers where they are usually managed and treated. Given the paucity of effective treatments available, the difficult access to clinical trials represents another relevant issue. The opening of new sponsored, multicenter, early-phase (Phases I–II) clinical studies previously scheduled at our institutions has been postponed, as well as the recruitment into the ongoing ones has been placed on hold, denying our patients a potentially effective therapeutic option. Finally, the drug-to-drug interactions between antiepileptic drugs and antiviral agents or chloroquine derivates, often used as empirical therapy for COVID-19 infection, potentially leading to toxic effects, represent a new concern that we faced. At the time of writing, in northern Italy, pressure on hospitals and intensive care units has sharply declined, while the contagion curve has settled on a plateau with a low number of newly infected cases. As neuro-oncologists working in three Institutions known as national referring hubs for the management of PBTs (Humanitas Cancer Center, Milan; Department of Medical Oncology, Bellaria Hospital, Bologna; and Veneto Institute of Oncology, Padua) and located frontline in the most affected endemic regions of Italy, we have directly experienced the dramatic impact of this pandemic. Moreover, two of the three centers being part of academic, general hospitals with an emergency department admitting SARS-CoV-2 positive subjects on a daily basis were forced to completely revolutionize their organization in just a few days, redefining spaces and reallocating medical resources (13). Several COVID-19 isolated wards have been created, and new intensive care beds have been built up wherever possible. Clinical activities not strictly necessary have been temporarily suspended and physicians working in these sectors redistributed. In our three institutions, oncologists continued to do their job full time giving care to their patients with the best intensity, limiting unnecessary hospital access and implementing COVID-free paths and all the safety procedures required to protect patients and medical staff. Now, thanks to the strict lockdown measures the biggest storm is moving away behind us and it is the right time to take a first balance, reflecting on the choices made and planning the future, with the concrete possibility of a second wave of infections in the next autumn. In the present paper, we would like to discuss some critical aspects involving management of PBT patients during the pandemic, sharing our personal experience on how we have modified our neuro-oncological daily practice to ensure either the safety and the continuum-of-care of our patients. We also present some preliminary data about the prevalence of the infection among the patient population of our three referral Neuro-Oncology Centers, showing the features and the clinical course of PBTs patients who got infected by SARS-CoV-2.

First of all, we have educated all our patients and their caregivers on the importance to strictly respect the correct behavior rules, as maintaining social distance, limit all unnecessary interactions, wearing mask, and frequent handwashing. Most of these recommendations have been given by phone/video contact or emails, and then reinforced directly during the onsite visits for those patients for whom it was necessary to come to the centers. We deferred all tumor assessment and clinical evaluations of asymptomatic or clinically stable, low-risk, PBT patients such as meningiomas and IDH-mutant low-grade glioma (WHO grade II). Taking decisions whether or not to start oncologic treatments must be carefully evaluated case-by-case, weighing potential risks of delaying and benefit gained for every proposed therapeutic intervention. For example, we considered reasonable and safe the postponing of adjuvant treatments (radiotherapy and chemotherapy) in the case of slow growth IDH-mutant low-grade gliomas, planning a new brain imaging in a 4- to 6-month period after surgery. We spared chemotherapy in likely non-responder patient population such as elderly or frail glioblastoma (GBM) patients with unmethylated MGMT promoter, as well as taking into account an hypofractionated radiotherapy approach to reduce the number of patients' access to the Hospital (14, 15). We avoided second or third line potentially immunosuppressive treatments in patients with poor performance status. Surgical indications were discussed in remote multidisciplinary tumor boards and avoided when the survival benefit expected is likely to be marginal. Due to the detrimental effects of inhibiting antiviral immunity and general immunosuppression, we carefully weighed the use of steroids, administering the lowest possible dose (ideally 10 mg of prednisone or equivalent that is an anti-inflammatory dose without immunosuppressive effects) for preventing or control brain edema and neurological symptoms (16). We limited the length of corticosteroid treatment to the shortest period of time, planning a fast tapering after clinical improvement. In patients taking high-dose steroids, we implemented prophylaxis with trimethoprim/sulfamethoxazole to reduce the risk of development of other interstitial pneumonias, and the concomitant use of low-dose diuretics with a steroid-sparing effect, such as furosemide. For those patients, in the case of onset of respiratory symptoms, we recommended performing a chest CT scan, even in the absence of fever. Given the aforementioned difficulties to travel and reach referral centers, we empowered cooperation with local institutions often lacking a particular expertise in these rare tumors and territorial primary care to ensure continuum-of-care in patients receiving active treatments. We ensured case-by-case direct communication with local physicians giving them all the necessary support to manage both cancer treatments and supportive care, including antiepileptic drugs. We implemented the use of direct phone calls, email contacts, or telemedicine with patients to check results of blood tests or give guidance on adverse events or disease-related symptom management. All our three centers followed the same SARS-CoV-2 testing policy. We tested all patients before hospitalization, outpatients with fever or respiratory symptoms before or during any active anticancer treatment, or patients with family members or caregivers with a known positivity for SARS-CoV-2. If a patient without symptoms or with mild symptoms tests positive, we can evaluate starting or continuing an oncologic treatment only after at least 1 month and the negativity of two nasopharyngeal swab for polymerase chain reaction (PCR) analysis of SARS-COV-2 performed 24 h apart. Neuro-oncologists must play a crucial role even in the multidisciplinary management of COVID-19-positive PBT patients during an eventual hospitalization, supporting pulmonologists, intensivists, and other specialists in clinical decision making. Indeed, we should help our colleagues in defining prognosis of our patients and accordingly the indication to invasive respiratory support and resuscitation. Finally, given the inability to access the COVID-19 wards, we took charge of communication with close relatives, keeping them constantly informed about patients' clinical conditions and the course of hospitalization. Among approximately 800 patients referring to our three centers between the end of February and the end of May, we reported a total of only five cases of SARS-CoV-2 infection assessed through the positivity of nasopharyngeal swab or bronchoalveolar lavage: four patients have a diagnosis of IDH wild-type GBM, one of IDH-mutant anaplastic astrocytoma. All patients infected were male, with a median age of 54 (range: 42–64), and were treated in Lombardy and Veneto. At the time of positivity to the test, the median dose of steroids assumed was 4 mg of dexamethasone (range: 2–16), and in three cases, the total lymphocyte count was ≤500/μL. Two patients were on active third-line treatment both with a metronomic temozolomide schedule (100 mg/m^2^, 3 weeks on and 1 week off), two were diagnosed with COVID-19 after an interruption of their second-line treatment with regorafenib, and one just came out of a second-surgery, and a re-radiation had been scheduled. In four out of five cases, presentation symptoms were fever, with respiratory insufficiency and radiological evidence of interstitial pneumonia, whereas only one patient was asymptomatic at the time of diagnosis. Demographic and clinical features of our patients infected by SARS-CoV-2 are summarized in Table 1.

**Table 1:** Brain tumor patients infected by SARS-CoV-2.

**Case**	Age*(years)	**Sex**	**Histology and molecular profile**	**Last treatment (A/C)**	Lymphocyte count* (×10^**3**^/mm^**3**^)	Steroid Therapy* (DEX mg dose)	**SARS-CoV-2 diagnosis (NFS/BAL)**	**COVID-19Clinical manifestations**
1	44	male	IDH wild-type GBM	Second surgery (C)	2.3	YES(2 mg)	NFS	NOasymptomatic
2	54	male	IDH wild-type GBM	metronomic Temozolomide (A)	0.4	YES(8 mg)	BAL	YESinterstitial pneumonia
3	43	male	IDH mutant astrocytoma	metronomic Temozolomide (A)	0.1	YES(16 mg)	NFS	YESinterstitial pneumonia
4	56	male	IDH wild-type GBM	Regorafenib (C)	0.5	YES(4 mg)	NFS	YESinterstitial pneumonia
5	64	male	IDH wild-type GBM	Regorafenib (C)	1.43	YES(2 mg)	NFS	YESinterstitial pneumonia

*A, active; BAL, bronchoalveolar lavage; C, concluded; DEX, dexamethasone; GBM, glioblastoma; IDH, isocitrate dehydrogenase; NFS, nasopharyngeal swab*.

* *At the time of SARS-CoV-2 infection*.

Three patients died due to COVID-19-related pneumonia; one patient recovered from the infection but was unable to continue oncological treatments due to the severe worsening of his clinical conditions, while the last one, after 1 month in home isolation and two consecutive negative swabs received a second radiation therapy (25 Gy in five fractions). Interestingly, four out of five cases experienced a significant worsening of their neurological status concurrently with the development of typical COVID-19 symptoms, despite the evidence of stability by brain MRI. This observation may reinforce the hypothesis of a potential direct CNS involvement by SARS-COV-2 causing a neurological deterioration not dependent on brain tumor progression.

Other international cooperative groups of experts proposed their insights and recommendations on neuro-oncological patients' management amid the COVID-19 outbreak (17, 18). This sort of guidelines reflect most of the actions that we effectively took during the peak of infections, being in some parts even more severe and stringent. Now that we are in a post-emergency phase, with the Italian lockdown recently ended, we can look back at our experience trying to assess the real impact of COVID-19 outbreak on our neuro-oncological community. Given the likely presence of asymptomatic cases or patients already in supportive care who died in long-term care facilities who were never tested, it will be really difficult to define the exact prevalence of the infection among our patients. However, the small number of cases who had severe or fatal complications from the SARS-COV-2 infection in our heavily hit regions somehow reassures us and shows the sustainability of carrying out a standard neuro-oncology practice from now on.

Since we currently do not know when this pandemic will end, or if the pathogen spread could even be controlled through a vaccination strategy or effective antiviral drugs, it is imperative to focus all our efforts on implementing safe COVID-free pathways into our centers that can guarantee the full safety of patients and staff. In this complex and dynamic scenario, where at least initially the coexistence with the SARS-CoV-2 will be obliged, we strive to not abandon the intensity of care for our PBT patients.

## Author Contributions

All authors equally contributed to the conception and writing of the present manuscript.

## Conflict of Interest

The authors declare that the research was conducted in the absence of any commercial or financial relationships that could be construed as a potential conflict of interest.

## References

[1] Johns Hopkins University & Medicine Coronavirus COVID-19 Global Cases. Coronavirus Resource Center (2020). Available online at: https://coronavirus.jhu.edu/map.html (accessed August 10, 2010).

[2] Remuzzi A Remuzzi G. COVID-19 and Italy: what next? Lancet. (2020) 395:1225–8. 10.1016/S0140-6736(20)30627-9 32178769 PMC7102589

[3] Brandes AA Di Nunno V. How to face cancer treatment in the COVID-19 era. Expert Rev Anticancer Ther. (2020) 20:1–4. 10.1080/14737140.2020.1766355 32370563

[4] Wang H Zhang L Risk of COVID-19 for patients with cancer. Lancet Oncol. (2020) 21:e181 10.1016/S1470-2045(20)30149-2 32142621 PMC7129735

[5] Kuderer NM Choueiri TK Shah DP Shyr Y Rubinstein SM Rivera DR. Clinical impact of COVID-19 on patients with cancer (CCC19): a cohort study. Lancet. (2020) 395:1907–18. 10.1016/S0140-6736(20)31187-9 32473681 PMC7255743

[6] Wise J. Covid-19: cancer mortality could rise at least 20% because of pandemic, study finds. BMJ. (2020) 369:m1735. 10.1136/bmj.m1735 32349991

[7] Pinato DJ Zambelli A Aguilar-Company J Bower M Sng C Salazar R. Clinical portrait of the SARS-CoV-2 epidemic in European cancer patients. Cancer Discov. (2020):CD-20-0773. 10.1016/j.annonc.2020.08.1743. [Epub ahead of print]. PMC7668225 32737082

[8] Grabowski MM Sankey EW Ryan KJ Chongsathidkiet P Lorrey SJ Wilkinson DS Immune suppression in gliomas. J Neurooncol. (2020). 10.1007/s11060-020-03483-y. [Epub ahead of print]. PMC7843555 32542437

[9] Dai M Liu D Liu M Zhou F Li G Chen Z. Neurologic manifestations of hospitalized patients with coronavirus disease 2019 in Wuhan, China. JAMA Neurol. (2020) 77:683–90. 10.1001/jamaneurol.2020.1127 32275288 PMC7149362

[10] Cardona GC Quintana Pájaro LD Quintero Marzola ID Villegas YR Moscote Salazar LR. Neurotropism of SARS-CoV 2: mechanisms and manifestations. J Neurol Sci. (2020) 412:116824. 10.1016/j.jns.2020.116824 32299010 PMC7141641

[11] Politi LS Salsano E Grimaldi M. Magnetic resonance imaging alteration of the brain in a patient with coronavirus disease 2019 (COVID-19) and anosmia. JAMA Neurol. (2020) 77:1028–9. 10.1001/jamaneurol.2020.2125 32469400

[12] Baig AM Khaleeq A Ali U Syeda H. Evidence of the COVID-19 virus targeting the CNS: tissue distribution, host-virus interaction, and proposed neurotropic mechanisms. ACS Chem Neurosci. (2020) 11:995–8. 10.1021/acschemneuro.0c00122 32167747

[13] Dipasquale A Persico P Lorenzi E Bertossi M Santoro A Simonelli M. Conducting phase I trials during the SARS-Coronavirus-2 outbreak: about science and care. Front Oncol. (2020) 10:926. 10.3389/fonc.2020.00926 32574280 PMC7256652

[14] Weller M van den Bent M Tonn JC Stupp R Preusser M Cohen-Jonathan-Moyal E. European Association for Neuro-Oncology (EANO) Task Force on Gliomas. European Association for Neuro-Oncology (EANO) guideline on the diagnosis and treatment of adult astrocytic and oligodendroglial gliomas. Lancet Oncol. (2017) 18:e315–29. 10.1016/S1470-2045(17)30194-8 28483413

[15] Ramakrishna R Zadeh G Sheehan JP Aghi MK. Inpatient and outpatient case prioritization for patients with neuro-oncologic disease amid the COVID-19 pandemic: general guidance for neuro-oncology practitioners from the AANS/CNS Tumor Section and Society for Neuro-Oncology. J Neurooncol. (2020) 147:525–9. 10.1007/s11060-020-03488-7 32274630 PMC7145274

[16] Dixit KS Kumthekar PU. Optimal management of corticosteroids in patients with intracranial malignancies. Curr Treat Options Oncol. (2020) 21:77. 10.1007/s11864-020-00771-7 32734428

[17] Bernhardt D Wick W Weiss SE Sahgal A Lo SS Suh JH. Neuro-oncology management during the COVID-19 pandemic with a focus on WHO grade III and IV gliomas. Neuro Oncol. (2020) 22:928–35. 10.1093/neuonc/noaa113 32369601 PMC7239150

[18] Mohile NA Blakeley JO Gatson NTN Hottinger AF Lassman AB Ney DE. Urgent considerations for the neuro-oncologic treatment of patients with gliomas during the COVID-19 pandemic. Neuro Oncol. (2020) 22:912–7. 10.1093/neuonc/noaa090 32277236 PMC7184330

